# The Practical Medicine Series

**Published:** 1915-06

**Authors:** A. E. Sweatland

**Affiliations:** Nacogdoche, Texas


THE PRACTICAL MEDICINE SERIES.—OBSTETRICS. By Joseph B. DeLee, A. M., M. D., with the collaboration of Herbert M. Stowe, M. D., Series, 1914. The Year-Book Publishers, Chicago. Price, $1.35.

The value of such a review to the general practitioner can scarcely be estimated. To the man in active field work, time is very valuable, and the more to the point he can have his reference volumes the better. This little work is all that could be desired in this respect. The subject is a very important one to every practitioner, and especially to the one who is doing general work. The volume covers the whole field in review, in a readily acceptable manner, and its timely teaching should be the means of saving life.

The author has very tersely called attention to errors in the management in given cases, and has, on the other hand, been just as free to commend that which to him, from a large experience, seems best. The section on Sepsis in the Puerperal State is in itself worth many times the price of the volume.

We may, and rightfully, differ in the management of this class of cases, but it would be difficult to conceive how more of value could be crowded into the same space, with so little, that is not valuable.

The book work is good enough.

Nacogdoches, Texas. A. E. SWEATLAND.

